# Disposition effect and reference points: An experimental study

**DOI:** 10.1371/journal.pone.0284171

**Published:** 2023-04-12

**Authors:** Newton da Costa, Ana Luiza Paraboni, Marco Goulart

**Affiliations:** 1 Graduate Program in Administration, School of Business, Pontifical Catholic University of Paraná, Curitiba, Brazil; 2 Department of Business Administration, Federal University of Santa Catarina, Florianópolis, Brazil; 3 Department of Production Engineering, Federal University of Santa Catarina, Florianópolis, Brazil; University of Durham: Durham University, UNITED KINGDOM

## Abstract

This study calls into question the default computation of the disposition effect that uses the average purchase price as a reference point. We show, through a lab experiment, that the reference price of participants can change depending on the experimental behavioral design that is used. Our results show that for the control group, different reference prices do not show significant differences in the computation of the disposition effect, thus supporting the use of the average purchase price as a reference. However, this is not the case for participants in the treatment group. With the addition of experimental treatment concerning the disclosure of the final balance of the participants, the reference prices to compute the disposition effect showed statistically significant differences. The need to display their results caused these participants to use the first purchase price as a reference point when selling their assets.

## Introduction

The behavioral finance literature has reported the existence of several anomalies in investor decision making. One of the most frequently studied anomalies is the disposition effect, which consists of the fact that investors tend to hold losing positions longer than gaining positions. The term "disposition effect" was coined by Shefrin and Statman [1] when they analyzed the percentages of gains and losses in mutual fund transactions in the USA. Another work, among the most commonly cited on the subject, is that of Odean [2], who analyzed the operations of clients of a discount brokerage house, also in the USA. Odean [2] proposed one of the most widely used methods for computing the disposition effect, both for investors trading in the real stock market and for participants in experimental settings.

The disposition effect has been related to some investor characteristics. There is evidence that it is lower when financial advice has been given [3], and when investors are experienced [4], and that it is higher among inexperienced investors [5–7], and in women, and people from certain cultures [8].

In his paper, Odean [2] reported that before adopting the average purchase price of a stock as a proxy for the true reference point in the computation of the disposition effect, he analyzed three other possible reference prices: the highest purchase price, the first purchase price, and the most recent purchase price. He found that the four forms of computation showed statistically similar results, and opted to use the average purchase price as the reference. It should also be mentioned that Weber and Camerer [9] and Rau [10], in experimental settings, also found no differences in computing the disposition effect with either FIFO or LIFO accounting principles.

Based on the findings of Odean [2], numerous studies on the disposition effect have used the average purchase price as the reference point [11–17]. More recently, Brettschneider, Burro and Henderson [18] argued that the average purchase price is the most natural term of comparison and also the appropriate benchmark from an accounting perspective. However, the classification of assets into winners or losers considering only average purchase prices may not actually reflect the psychological processes in investors’ minds, as it would be counterintuitive to assume that investors facing a sell decision are not influenced by stock prices at any time other than when they purchased the stock.

Although Odean [2] suggested that the average purchase price shows similar results to the other methods, little is known about the influence that experimental designs can have on the outcome of the disposition effect (its statistical significance), since its level depends on the reference points adopted. In Odean’s [2] study, real data from the financial market were used. In this paper, we investigate whether the average purchase price shows similar results to other reference prices in an experimental design.

The present study was conducted based on a computational investment simulation applied to undergraduate students. The investment simulation follows the methodology proposed by Weber and Camerer [9] and Goulart et al. [19]. While Odean [2] used real data for the evaluation of the disposition effect, we chose to use an experiment due to the greater control it enables over the variables studied. We point out that the discussion on using the average price to compute the disposition effect is also present in laboratory experiment studies [10,20].

In our experiment, following the methodology used by Goulart et al. [19], participants were randomly assigned to two groups: public and private. In the public group, students were invited to perform the computer simulation and make their results public at the end of the experimental session, and a ranking with the positions of all the participants was publicly disclosed. In the private group, the individuals kept their results confidential.

Heimer [21] showed that individuals almost double their level of disposition effect when they begin to participate in online social networks linked to investments. The author noted that concerns over self-image or reputation significantly influence the disposition effect, because the appearance of success allows for a more socially persuasive interaction with others. Likewise, according to Kadous et al. [22], investors are driven by the need to maintain a positive self-image. Investors recognize the position of loss as threatening and they seek to defend themselves against the threat to their self-image precisely by delaying the recognition of losses.

When individuals are in an environment where their actions are observed in real time and their unsatisfactory performance can tarnish their reputation, they become more aware of the negative consequences associated with poor performance. Therefore, they avoid showing bad decisions, limiting losses instead of accepting them and looking for bigger gains in order to show their superior negotiation skills [23].

Ashraf et al. [24] conducted a natural field experiment to separately identify channels through which awards can affect behavior, disaggregating the effect of social comparison through the disclosure (private or public) of ranking information from the effect of employer recognition and social visibility. The results of the study suggested that awards may have a negative effect on performance as they facilitate social comparison, although they still have a positive effect through employer recognition and increased social visibility.

In the same line of reasoning, the study of Bault et al. [25] suggested that humans have a strong preference for higher positions in social ranking, and this behavior influences how they evaluate outcomes and make choices. In their experiment, the participants chose between lotteries with different levels of risk. They found that the weight of gains and losses differed in the private and social domains. For private outcomes, the weight of losses loomed larger than gains, while in the social domain, the weight of gains loomed larger than losses. The study found that the larger weight assigned to social gains affected choices, leading to more risky and dominant behavior towards weaker competitors.

Futhermore, Goulart et al. [19] demonstrated that the simple expectation of individuals having to reveal and compare their financial performance with that of others causes the disposition effect to increase, driven mainly by the greater tendency to sell shares that have increased in value in relation to the purchase price. Among the various possible reference points, the first purchase price would be the easiest to remember and focus on in a stressful situation. It would be like a decision associated with System 1 of thinking, with less energy expenditure, a more automatic decision and driven by emotions and associations [26–28].

We emphasize that our goal in this paper is not to propose a method that enables us to know in advance which reference point should be used under a given experimental manipulation. Nor is it our objective to encourage a reference to be used by the individual or to detect which price was used in the decision-making process. As mentioned by [18], this type of investigation is limited by the fact that the data do not tell us whether investors are choosing alternative reference points. However, it is fundamental to observe how strongly the disposition changes. This will allow an investigation of how psychological processes related to the attention paid to the trajectory of stock prices can influence sales decisions, as well as an investigation of possible underlying cognitive patterns.

Our goal is simpler: to show that, depending on the experimental environment, the use of the average price as a reference in calculating the disposition effect can be misleading.

Our results showed that, for the control group (private group), different reference prices did not present significant differences in the computation of the disposition effect, thus corroborating the use of the average purchase price as a reference.

However, this was not the case for the participants in the treatment group. In this group, the disposition effect averages showed statistical differences according to the reference points used. We highlight the first purchase price because it has the highest average in the group and, being the only significant one according to the multiple linear regression, indicated a greater disproportion of sales with gains than with losses in relation to the other reference points.

These results are relevant because they show the importance that studies on the disposition effect, especially those where experimental manipulations occur, should evaluate the effect according to different reference points. The most common practice that has been adopted in most studies, whether experimental or not, is to use only the average purchase price, possibly due to the prior understanding that other reference prices would show similar results [2]. In this study, we show that using only the average price can attenuate the level of biased behavior found by the researchers, as in the treatment condition, the first purchase price showed higher means of the disposition effect.

This paper is organized as follows. Section 2 presents the most common way of computing the disposition effect, which is the one based on Odean [2], and the different possibilities of reference points for the purchase price. Section 3 details the design of the experiment used in this study. Section 4 presents the results and discussion, and Section 5 concludes the study.

### Investment simulator and measurements of the disposition effect

**Fig 1:**
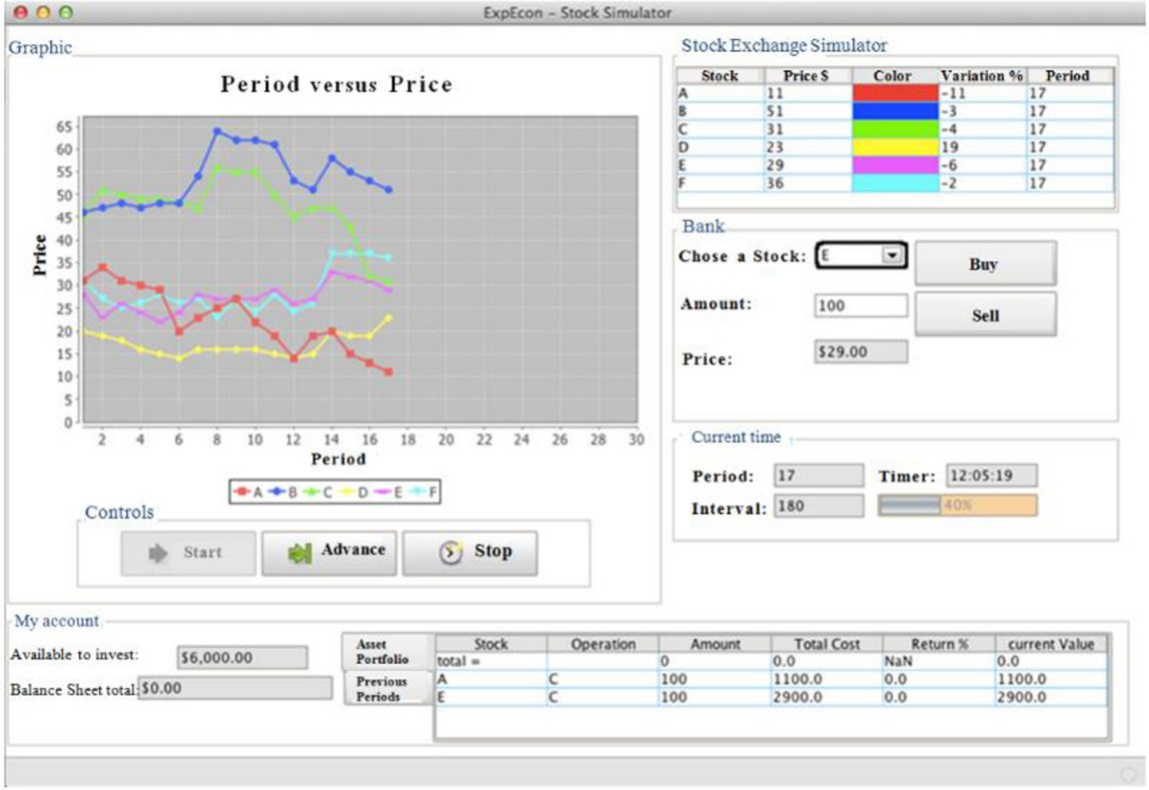
ExpEcon investment simulator’s main interface.

This research is based on the experimental designs of Weber and Camerer [9] and Goulart et al. [19]. We attempted to understand the behavior of individuals in relation to the cognitive bias called the disposition effect, which we measured from different reference points. For this purpose, the data for this research were collected in an investment simulation software called ExpEcon. It is hosted in the Github repository and can be accessed at https://github.com/schmaedech/expecon. Fig 1 shows the main screen of the ExpEcon software.

ExpEcon is an exogenous investment simulator that has six stocks to trade: A, B, C, D, E, and F. The prices of these stocks can be generated randomly, as in Weber and Camerer [9], or based on the stocks that made up Ibovespa in previous periods. Ibovespa is the market index with the most liquid stocks in the Brazilian stock market.

The window called "Stock Exchange Simulator" shows the participant the last period’s information for each of the six stocks. This information includes the price, color (for viewing on the graph on the left), price variation in relation to the previous period, and information period.

The window named "Period x Price" presents a chart for the participant to visualize the price of the stock in each period and, to facilitate viewing, each stock has a different color. Besides the color, the assets also have a symbol to identify them, which allows colorblind individuals to participate in the study. Thus, as time goes by, the chart is updated with information from the previous period.

The window called "bank" is where the participant effectively conducts his buying and selling operations, chooses the asset to be traded and indicates the desired value. ExpEcon does not allow short selling or financing.

The window called "current time" is where the participant can see the interval between periods (in seconds) and how much time is left before the end of the period (when the bar is full, the software automatically switches to the next period). ExpEcon simulates 30 sub-periods (in the case of this study), and the total simulation time varies by participant, reaching a maximum time of 90 minutes (up to three minutes for each of the 30 sub-periods). In the first three rounds, the participants only observe the oscillations to attempt to identify any possible trends. From the fourth period onwards, the participants are free to start buying and selling assets.

In addition, the window called "my account" shows the amount of available funds (free for trading) and the current value of the portfolio added to the cash on hand (total balance sheet). On the right side, we see the participant’s asset portfolio, all the transactions carried out per asset, the amounts traded, the lot cost of the share units, the current value of the shares in the portfolio, and the percentage difference between the current value and the lot cost. In this study, all the participants began the simulation with 10,000 currency units to trade.

The simulation does not provide any information other than the current and past prices of each stock. The software also generates an output file with a report of all asset purchases and sale transactions in each period simulated by the participant.

The main variable of interest in this study is the coefficient associated with the disposition effect of a participant in the experiment. For this purpose, the measure proposed by Odean [2] was used, which measures the difference between the proportion of gains and the proportion of losses of each participant. Thus, the disposition effect is calculated as follows:

$$DE_{i}=\frac{N_{GR}^{i}}{N_{GR}^{i}+N_{GP}^{i}}-\frac{N_{LR}^{i}}{N_{LR}^{i}+N_{LP}^{i}}$$ (1)

Where *DE*_*i*_ is the disposition coefficient of individual i; $N_{GR}^{i}(N_{LR}^{i})$ is the number of trades of investor i with a realized gain (loss) and $N_{GP}^{i}(N_{LP}^{i})$ is the number of potential trades for investor i with a gain (loss). The coefficient varies from -1 to +1, where *DE*_*i*_ = 1 means that the individual made only sales at a profit and *DE*_*i*_ = -1 means that the individual made only sales at a loss. *DE*_*i*_ = 0 means that the individual has no disposition effect.

A sale is defined as a winner (loser) if the sale price is higher (lower) than the reference price. Odean [2] reported that the average purchase price, the maximum purchase price, the first purchase price and the last purchase price are all statistically similar when it comes to the actual buying and selling transactions by clients of a US discount brokerage firm. In our experimental study, in keeping with our objective, we looked at five possible reference points: average purchase price (DE_reg), minimum purchase price (DE_minbuy), maximum purchase price (DE_maxbuy), first purchase price (DE_firstbuy) and last purchase price (DE_lastbuy).

As we have already defined, the disposition effect is related to the tendency to realize gains quickly and postpone realizing losses. Therefore, to detect whether the computed coefficient associated with the disposition effect is statistically significant, Odean [2] suggested the test of difference of means with the following null hypothesis: the number of sales with a gain is equal to the number of sales with a loss. Thus, according to Odean [2], *DE*_*i*_ can be evaluated by the t-statistic

$$t=\frac{PGR-PLR}{SE}$$ (2)

where PGR is the proportion of gains realized, PLR is the proportion of losses realized, and the standard error SE is given by:

$$SE=\frac{s}{\sqrt{n}}$$ (3)

where S is the sample standard deviation and *n* is the sample size.

### Experiment design and hypotheses

The data used in the present experiment are taken from the PhD dissertation of Goulart [29] and Goulart et al. [19]. However, in this study, unlike from the previous ones, we computed the disposition effect based in five different reference points for each participant. The data for this study can be accessed at https://doi.org/10.6084/m9.figshare.17091719.v3

We made this computation change to highlight whether the context in which they are embedded can change the perspective of the reference points for the disposition effect. The design requirements of an experiment often mean that the selected samples are small, with greater internal validity, that is, the results can be attributed to the interventions. However, external validity will be more difficult to establish [30].

The experimental sessions for the students took place in the university computer lab, and participants were assigned to individual desktops and were not allowed to communicate with one another. The experimenter provided instructions at the beginning of each session. This environment can be considered suitable for the application of a simulation of computational investments, because, in addition to being isolated, students can be kept away from each other. The experiment was conducted in the second term of 2013.

The sample was composed of undergraduates enrolled in economics, accounting or management courses at the Federal University of Santa Catarina. The participants were randomly assigned to two groups: public (n = 62; 61.3% males) and private (n = 56; 60.7% males). Participants who did not make any sales during the period were excluded from the sample.

The first group was informed that they were required to reveal their results at the end of the simulation, in accordance with Goulart et al. [19]. Therefore, after the simulation ExpEcon was completed, the final balance of everyone was known by the entire group, as each participant had to go to the blackboard and write his/her name, final balance, and ranking among the participants of that experimental session, in descending order. This study was approved by the Research Ethics Committee of the University of Santa Catarina registered as Number 711.395.

According to Goulart et al. [19], the need to expose results may lead to biased behavior due to individuals’ strategic attempt to protect themselves from the embarrassment of ending the trading session at the bottom of the performance rating. It should be highlighted that the participants were clearly informed of this condition before beginning the simulation.

As for the private group, the participants performed the investment simulation, but at the end they were not required to reveal their results to their colleagues, and the financial results remained confidential.

All the individuals received a financial reward for participating in the study according to his/her final balance. 1,000 monetary units of the simulation was equal to a R$1 cash prize. For example, if at the end of the simulation the final balance, which appears on the simulation screen, was 15,000 monetary units, the subject received R$15.00 (equivalent to US$4.50 at the time of the experiment). To ensure the confidentiality of the result of the private group, at the end of the simulation, the participants received the amount corresponding to their final balance in an envelope.

Based on the above experiment design and bearing in mind what was discussed in the Introduction regarding the impact of performance exposure to peers, we sought to test the following research hypotheses:

Hypothesis 1: Under experimental conditions without treatment of the environment, the average purchase price is equivalent to the other reference points for the computation of the disposition effect.

Hypothesis 2: Under experimental conditions that stimulate additional emotions, the disposition effect coefficients show statistical differences according to the reference price.

## Results and discussion

As already pointed out, in this experiment, we randomly assigned the students to two different groups: the treatment group, which we called the "public" group (n = 62), and the control group (n = 56), which we called the "private" group. Table 1 presents the descriptive statistics of the disposition effect coefficients, as well as the test of difference of means to verify whether the calculated coefficients are significant.

**Table 1:** Descriptive statistics by treatment.

Disposition effect	Mean		Standard Deviation		*t* test (DE = 0)		p-value	
	Private	Public	Private	Public	Private	Public	Private	Public
DE_reg^1^	0.033	0.053	0.207	0.223	1.189	1.872	0.239	0.066^*^
DE_minbuy^2^	0.068	0.078	0.154	0.183	3.279	3.365	0.002^***^	0.001^***^
DE_maxbuy^3^	0.027	0.029	0.215	0.215	0.933	1.049	0.355	0.298
DE_firstbuy^4^	0.063	0.152	0.281	0.178	1.674	6.738	0.100	0.000^***^
DE_lastbuy^5^	0.046	0.111	0.181	0.248	1.913	3.515	0.061^*^	0.001^***^

Notes:

Private is the treatment where the subjects did not need to report their results (n = 56).

Public is the treatment where the subjects needed to report their results (n = 62).

^1^ Disposition effect with the average purchase price as a reference.

^2^ Disposition effect with the minimum purchase price as a reference.

^3^ Disposition effect with the maximum purchase price as a reference.

^4^ Disposition effect with the first purchase price as a reference.

^5^ Disposition effect with the last purchase price as a reference.

^***^significant at 1%;

^**^significant at 5%;

^*^significant at 10%.

The descriptive statistics for the private group revealed that only when considering the minimum price (p-value < 0.01), and the last price (p-value < 0.10) as a reference, did we find a significant disposition effect. In this case, the proportion of gains was statistically different from the proportion of losses. For the other points, there was no significant difference between the proportion of gains and losses realized. This result alone represents a weak point in the choice of reference price. When researchers analyze the disposition effect only by the average price, they may be missing information and drawing hasty conclusions about the biased behavior of individuals.

When it came to the group of students who were previously aware that they had to expose their financial results after the simulation, the subjects showed significant disposition effect for four of the reference prices tested: average purchase price (p-value < 0.10); minimum price; and first and last purchase price (p-value <0.01).

**Fig 2:**
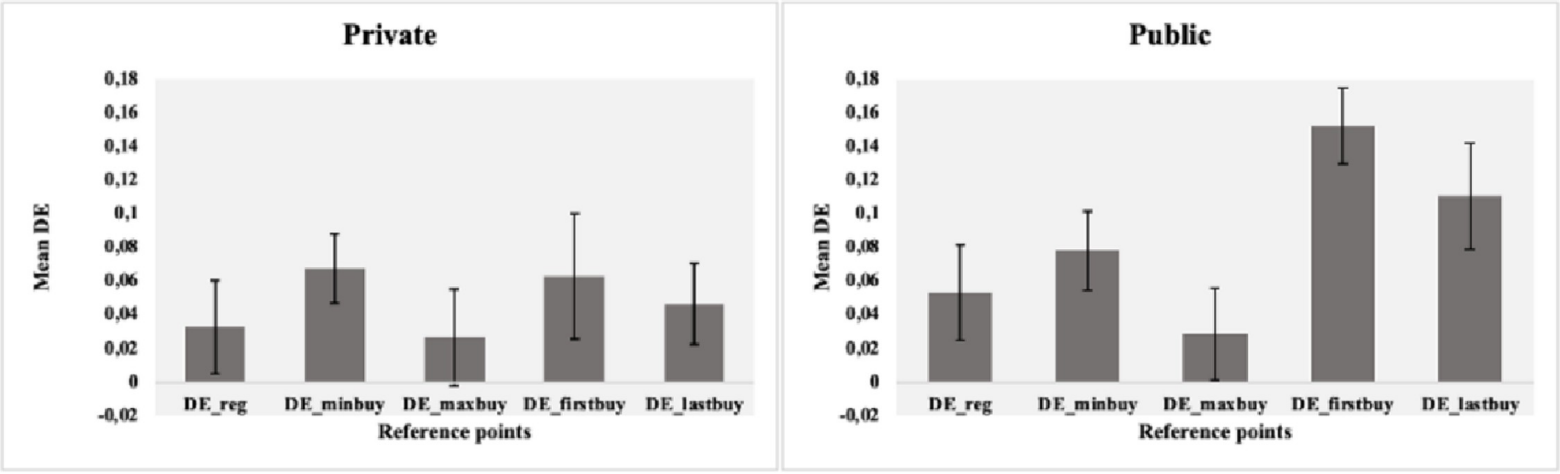
Graphical representation of the disposition effect coefficients, by treatment. Note: Error bars indicate the standard error of the mean.

Another result worth mentioning in the public group is the mean of the coefficients, with that of DE_firstbuy being 0.152, much higher than the other coefficients in the group. Fig 2 presents the averages by reference point and by treatment for a better visualization of the differences.

Fig 2 shows evidence that the coefficients may be different. However, next we present the results of the one-way ANOVA to understand whether the reference prices remain similar at the time when individuals need to report (or not) their results.

Table 2 shows that for the individuals who needed to expose their results after the simulation there was a significant difference between the means of the disposition coefficients calculated by the different reference points.

**Table 2:** ANOVA test.

Variables	Private		Public	
	Mean	F test [p-value]	Mean	F test [p-value]
DE_reg^1^	0.033	0.401 [0.808]	0.053	3.284 [0.012] ^***^
DE_minbuy^2^	0.068		0.078	
DE_maxbuy^3^	0.027		0.029	
DE_firstbuy^4^	0.063		0.152	
DE_lastbuy^5^	0.046		0.111	

Notes:

^1^ Disposition effect with the average purchase price as a reference.

^2^ Disposition effect with the minimum purchase price as a reference.

^3^ Disposition effect with the maximum purchase price as a reference.

^4^ Disposition effect with the first purchase price as a reference.

^5^ Disposition effect with the last purchase price as a reference.

^***^ significant at 1%.

However, in the private group, no significant differences were found in the coefficients, where there was no need for participants to expose their results to everyone. Consequently, we should emphasize that discussions about gains and losses and reference points need to be enhanced in the context of behavioral finance studies [31].

More important than checking whether there are differences between the coefficients is attempting to understand where these differences are found. To this end, we used the post-hoc test that lists the means, two by two, to then show which ones present statistical differences (Table 3). The principle of homoscedasticity of variances was met for both groups and, therefore, we used Tukey’s post-hoc test [32].

**Table 3:** Tukey’s post-hoc test, by treatment.

DE (I)	DE (J)	Private		Public	
		Mean difference (I-J)	p-value	Mean difference (I-J)	p-value
**DE_reg** ^**1**^	DE_minbuy	-0.035	0.908	-0.025	0.964
	DE_maxbuy	0.006	1.000	0.024	0.968
	DE_firstbuy	-0.030	0.945	-0.099	0.069^*^
	DE_lastbuy	-0.013	0.997	-0.058	0.551
**DE_minbuy** ^**2**^	DE_reg	0.035	0.908	0.025	0.964
	DE_maxbuy	0.041	0.846	0.049	0.689
	DE_firstbuy	0.005	1.000	-0.074	0.289
	DE_lastbuy	0.021	0.984	-0.032	0.912
**DE_maxbuy** ^**3**^	DE_reg	-0.006	1.000	-0.024	0.968
	DE_minbuy	-0.041	0.846	-0.049	0.689
	DE_firstbuy	-0.036	0.897	-0.124	0.011^**^
	DE_lastbuy	-0.020	0.988	-0.082	0.197
**DE_firstbuy** ^**4**^	DE_reg	0.030	0.945	0.099	0.069^*^
	DE_minbuy	-0.005	1.000	0.074	0.289
	DE_maxbuy	0.036	0.897	0.124	0.011^**^
	DE_lastbuy	0.016	0.994	0.042	0.806
**DE_lastbuy** ^**5**^	DE_reg	0.013	0.997	0.058	0.551
	DE_minbuy	-0.021	0.984	0.032	0.912
	DE_maxbuy	0.020	0.988	0.082	0.197
	DE_firstbuy	-0.016	0.994	-0.042	0.806

Notes:

^1^ Disposition effect with the average purchase price as a reference.

^2^ Disposition effect with the minimum purchase price as a reference.

^3^ Disposition effect with the maximum purchase price as a reference.

^4^ Disposition effect with the first purchase price as a reference.

^5^ Disposition effect with the last purchase price as a reference.

^**^ significant at 5%;

^*^ significant at 10%.

The first point that deserves to be highlighted is the statistical similarity between the reference prices in the private group. In this case, the reference prices were shown to be interchangeable, with no statistical differences among them.

On the other hand, when the participants needed to expose their results to the group, the disposition effect coefficient was higher when we used the first purchase price as a reference compared with the average purchase price and the maximum purchase price. Due to the statistical difference, this evidence leaves room for questions about the use of the average purchase price as a reference for the disposition effect, mainly because it is widely used in studies in the field, whether in studies with market data or with experimental data.

To corroborate the results in Table 3, we estimated a multiple linear regression model. We used the average purchase price, represented by the regression intercept, as a basis for comparison, and four dummies were included as independent variables. DE_minbuy is a dummy variable, where 1 means the reference point is the minimum purchase price and 0 any other reference point. For DE_maxbuy, 1 means the reference point is the maximum purchase price and 0 any other reference point. DE_firstbuy is also a dummy variable, where 1 represents the price of the first purchase and 0 represents any other reference point. Finally, for DE_lastbuy, 1 represents the last purchase price and 0 any other reference point. As a dependent variable, we used all the disposition coefficients calculated for each individual. The results are presented in Table 4.

**Table 4:** Multiple linear regression.

Variables^1^	Private		Public	
	Coefficient	p-value	Coefficient	p-value
DE_minbuy^2^	0.066	0.386	0.047	0.509
DE_maxbuy^3^	-0.012	0.879	-0.046	0.521
DE_firstbuy^4^	0.057	0.455	0.186	0.009^***^
DE_lastbuy^5^	0.026	0.737	0.108	0.13
Adj R^2^	-0.009		0.029	
F test	0.401		3.284	
p-value	0.808		0.012^**^	

Notes:

^1^ Disposition effect as dependent variable.

^2^ Dummy variable for reference point: (1) minimum purchase price and (0) other reference prices.

^3^ Dummy variable for reference point: (1) maximum purchase price and (0) other reference prices.

^4^ Dummy variable for reference point: (1) first purchase price and (0) other reference prices.

^5^ Dummy variable for reference point: (1) last purchase price and (0) other reference prices.

^***^ significant at 1%;

^**^ significant at 5%.

Confirming the results of Tukey’s test, Table 4 shows that the dummy variable representing the first purchase price had a significant impact on the dependent variable. The coefficient is positive and, therefore, when anchoring the price to the first purchase price of the stock, individuals in the public group showed even more biased behavior. In this case, the difference in the level of the disposition effect calculated when dealing with the two reference points (average price and first purchase price) is evident. These individuals sold significantly more shares in the gains domain than in the losses domain when the first purchase price was taken into account in the calculation of the disposition effect. For the private group, as expected, there was no significant impact.

Finally, based on the results reported above, if we were replicating the experiment of Goulart et al. [19] and comparing the DE of the public group with that of the private group, and using the average purchase price as a reference for both groups (see Tables 1 and 2), we would have to compare 0.053 with 0.033, which would give us 0.020 (p-value = 0.31). However, if we used the first price as a reference for the public group and the average purchase price for the private one, there would be a difference of 0.119 (= 0.152–0.033), with a p-value = 0.001, showing a significant difference between the DE of the two groups.

## Final considerations

One of the most documented biases in the field of behavioral finance is the so-called disposition effect, which refers to an individual’s greater propensity to sell a stock that has risen in value relative to a certain benchmark than a stock that has fallen in value. This study contributes to the literature on finance and the disposition effect bias by calling into question the default computation of this bias that uses the average purchase price as a reference point. We showed that the reference price of participants can change depending on the experimental design used.

In the control (private) group of our experiment, we observed that the five reference points used to compute the disposition effect were equivalent, corroborating our first research hypothesis. On the other hand, in our treatment (public) group, we showed that with the addition of experimental treatment concerning the disclosure (or not) of the individual financial performance of the simulation participants, the reference prices showed statistically significant differences, corroborating our second research hypothesis.

Little is yet known about the influence that experimental designs can have on the psychological processes of investors’ minds and their choice of reference points during their participation in an experiment and, consequently, on the computation of the disposition effect.

The main purpose of this study is to raise questions about the use of the average price as a reference when measuring the disposition effect. As the results have shown, in situations that instill extra emotions, the “default” calculation of this bias can be misleading. The exposure of results is only one of the factors surrounding investors, mainly because the number of new investors in the Brazilian capital market has increased significantly. In addition to this factor, we can mention others, such as the influence of positive/negative affections, investor experience in the capital market, and influence of market direction (upward or downward). In view of this, whether in a laboratory experiment or in the real market, we emphasize the need to estimate the disposition effect from each possible reference point and choose the most statistically appropriate one for the situation in question.

Furthermore, it remains an open question whether our analysis and its results could be transposed to the real financial market, where other variables could influence investor behavior and the choice of different reference points.
